# Effect of antiviral and immunomodulatory treatment on a cytokine profile in patients with COVID-19

**DOI:** 10.3389/fimmu.2023.1222170

**Published:** 2023-07-06

**Authors:** Diana Martonik, Anna Parfieniuk-Kowerda, Aleksandra Starosz, Kamil Grubczak, Marcin Moniuszko, Robert Flisiak

**Affiliations:** ^1^ Department of Infectious Diseases and Hepatology, Medical University of Bialystok, Bialystok, Poland; ^2^ Department of Regenerative Medicine and Immune Regulation, Medical University of Bialystok, Bialystok, Poland; ^3^ Department of Allergology and Internal Medicine, Medical University of Bialystok, Bialystok, Poland

**Keywords:** COVID-19, SARS-CoV-2, Th17 cells, CD4+ T cells, CD8+ T cells, cytokines, treatment

## Abstract

**Background:**

The severity of COVID-19 is associated with an elevated level of a variety of inflammatory mediators. Increasing evidence suggests that the Th17 response contributes to the severity of COVID-19 pneumonia, whereas Th22 response plays a regulatory role in SARS-CoV-2 infection. Two main types of available COVID-19 treatments are antivirals and immunomodulatory drugs; however, their effect on a cytokine profile is yet to be determined.

**Methods:**

This study aim to analyse a cytokine profile in peripheral blood from patients with COVID-19 (n=44) undergoing antiviral or/and immunomodulatory treatment and healthy controls (n=20). Circulating CD4+ and CD8+ T cells and their intracellular expression of IL-17A and IL-22 were assessed by flow cytometry.

**Results:**

Initial results showed an overexpression of IL-17F, IL-17A, CCL5/RANTES, GM-CSF, IL-4, IL-10, CXCL-10/IP-10 and IL-6 in COVID-19 patients compared to healthy controls. Treatment with remdesivir resulted in a significant decline in concentrations of IL-6, IL-10, IFN-alpha and CXCL10/IP-10. Immunomodulatory treatment contributed to a significant downregulation of IL-10, IFN-alpha, CXCL10/IP-10 and B7-H3 as well as upregulation of IL-22 and IL-1 beta. A combination of an antiviral and immunomodulatory treatment resulted in a significant decrease in IL-17F, IL-10, IFN-alpha, CXCL10/IP-10 and B7-H3 levels as well as an increase in IL-17A and IL-1 beta. We found significantly higher percentage of both CD4+ and CD8+ T cells producing IL-17A and CD4+ T cells producing IL-22 in patients with COVID-19.

**Conclusion:**

Administration of antiviral or/and immunomodulatory treatment resulted in a significant downregulation of pro-inflammatory cytokine expression and an upregulation of T cell absolute counts in most cases, thus showing effectiveness of treatment in COVID-19. SARS-CoV-2 infection induced cytokine overexpression in hospitalized patients with COVID-19 as well as lymphopenia, particularly a decrease in CD4+ and CD8+ T cell counts. Moreover, despite the reduced counts of CD4+ and CD8+ T cells, both subsets showed overactivation and increased expression of IL-17A and IL-22, thus targeting Th17 response might alleviate inflammatory response in severe disease.

## 1 Introduction

At the end of 2019, an unprecedented epidemic of pneumonia caused by a novel beta coronavirus, termed SARS-CoV-2 afterward, emerged in Wuhan (China) (1). The rapid spread of the virus worldwide resulted in global pandemic of the subsequent disease COVID-19 announced by WHO on 11 March 2020. Most people infected with SARS-CoV-2 experience mild to moderate respiratory disease that resolves on its own. However, elderly people and those burdened with comorbidities such as chronic respiratory disease, cardiovascular disease, diabetes, and cancer are more likely to develop severe COVID-19 (2).

Cytokine release syndrome (CRS), which is an excessive response to the production of pro-inflammatory cytokines and chemokines, may be responsible for the severe course of the disease according to research. Unusually high release of cytokines triggering immunopathological reaction is described as “cytokine storm” (1, 2). Studies have shown that the severity of COVID-19 is associated with an elevated level of inflammatory mediators such as tumor necrosis factor (TNF), CXC-chemokine ligand 10 (CXCL-10), monocyte chemoattractant protein 1 (MCP-1), interleukin (IL)-2, IL-6, IL-7, and IL-10. Elevated concentration of IL-6 has been shown to strongly correlate with COVID-19 mortality (3, 4). Aberrant release of pro-inflammatory mediators leads to apoptosis of lung epithelial and endothelial cells causing damage to the microvascular and alveolar epithelial cell barrier which leads to vascular leakage, alveolar oedema, and hypoxia (5). There is evidence that both Th17 and Th22 response can play an immunomodulatory role in some diseases, including viral infections. It has been shown that in some situations Th17 and Th22 can exert opposite immune effects. Increasing evidence suggests that the Th17 response contributes to the severity of COVID-19 pneumonia, whereas Th22 response plays a regulatory role in SARS-CoV-2 infection (6, 7).

There are two main types of available COVID-19 treatments – antivirals and immunomodulatory drugs. Remdesivir, an antiviral medication, has received significant recognition for its capacity to control and regulate the viral load and has been approved for the treatment of patients with COVID-19. It is a broad-spectrum nucleoside analogue that can target variety of single-stranded RNA viruses, including coronaviruses. Its primary mechanism of action is the inhibition of RNA-dependent RNA polymerase thus leading to the suppression of SARS-CoV-2 replication in respiratory-associated epithelial cells (8, 9). Immunomodulatory agents modify the response of the immune system by activating, inhibiting, or modulating various immune system components. Tocilizumab is a recombinant humanized monoclonal antibody that binds to IL-6 receptors to exert immunosuppressive effects and attenuate IL-6 activity. It was approved for treatment of the CRS in severe COVID-19 (9, 10). Glucocorticoids (GCs) are recommended for the treatment of oxygen-requiring patients with COVID-19. GCs exhibit pharmacologic effect through classic genomic mechanisms. Some immunosuppressive effects are based on the induction of gene transcription and synthesis of NF-kB protein inhibitors, resulting in inhibition of the synthesis of downstream proteins such as IL-1 and IL-6. GCs reduce the activation, proliferation, and survival of both T cells and macrophages. The dosage and timing of administration of GCs have a significant impact on the outcome of the critically ill patients. The initiation of the immune defense mechanism is inhibited by the premature administration of GCs, which raises the viral load and ultimately has negative consequences. Therefore, GCs are mainly used to treat critically ill patients with CRS (10, 11).

In this study, we performed an evaluation of immunological features of patients with COVID-19 hospitalized in the Department of Infectious Diseases and Hepatology at the Medical University of Bialystok Clinical Hospital, who were treated with tocilizumab, glucocorticoids and/or remdesivir. We aimed to assess the effect of treatment on a cytokine and T cell profiles, including the intracellular expression of IL-17A and IL-22. We showed that administration of antiviral or/and immunomodulatory treatment resulted in a significant downregulation of pro-inflammatory cytokine expression and an upregulation of T cell absolute counts in most cases, thus showing effectiveness of treatment in COVID-19.

## 2 Materials and methods

### 2.1 Study population

The study included 44 patients with COVID-19 hospitalized in the Department of Infectious Diseases and Hepatology at the Medical University of Bialystok Clinical Hospital in January to May 2021, who were treated with tocilizumab, glucocorticoids and/or remdesivir. SARS-CoV-2 infection was confirmed with reverse transcription polymerase chain reaction (RT-PCR) in a certified diagnostic facility before hospital admission. In addition, 20 volunteers vaccinated against SARS-CoV-2 with no inflammatory or autoimmune disease were enrolled as a control group.

Vital signs included in Modified Early Warning Score (MEWS) (oxygen saturation, heart rate, blood pressure, respiratory rate, body temperature, alertness, diuresis), full blood count, coagulation parameters, acute-phase proteins, and other tests necessary for proper care in accordance with good clinical practice were measured before and during treatment. MEWS score allows to stratify patients according to their clinical condition:

1. Asymptomatic or mild type
2. Stable patients with respiratory and/or systemic symptoms
3. Clinically unstable patients with respiratory failure
4. Patient in critical condition (acute respiratory distress syndrome)

Patients with asymptomatic/mild disease and patients in whom inflammatory changes in the lungs were not confirmed by imaging studies were excluded from the study. Additional exclusion criteria were the presence of autoimmune diseases, malignancy, and current immunomodulatory treatment for diseases other than COVID-19. Management and treatment followed current national recommendations for COVID-19 (12, 13).

Remdesivir was administered to patients admitted to the hospital during first week from the onset of symptoms, dexamethasone was given to patients receiving antiviral drug, who also required oxygen therapy and tocilizumab was administered to patients with IL-6 concentration above 100 pg/mL. Treatment was classified as follows: a) antiviral therapy – remdesivir; b) immunomodulatory therapy – tocilizumab or/and dexamethasone; c) mixed therapy – combination of antiviral and immunomodulatory treatment.

**Figure 1:**
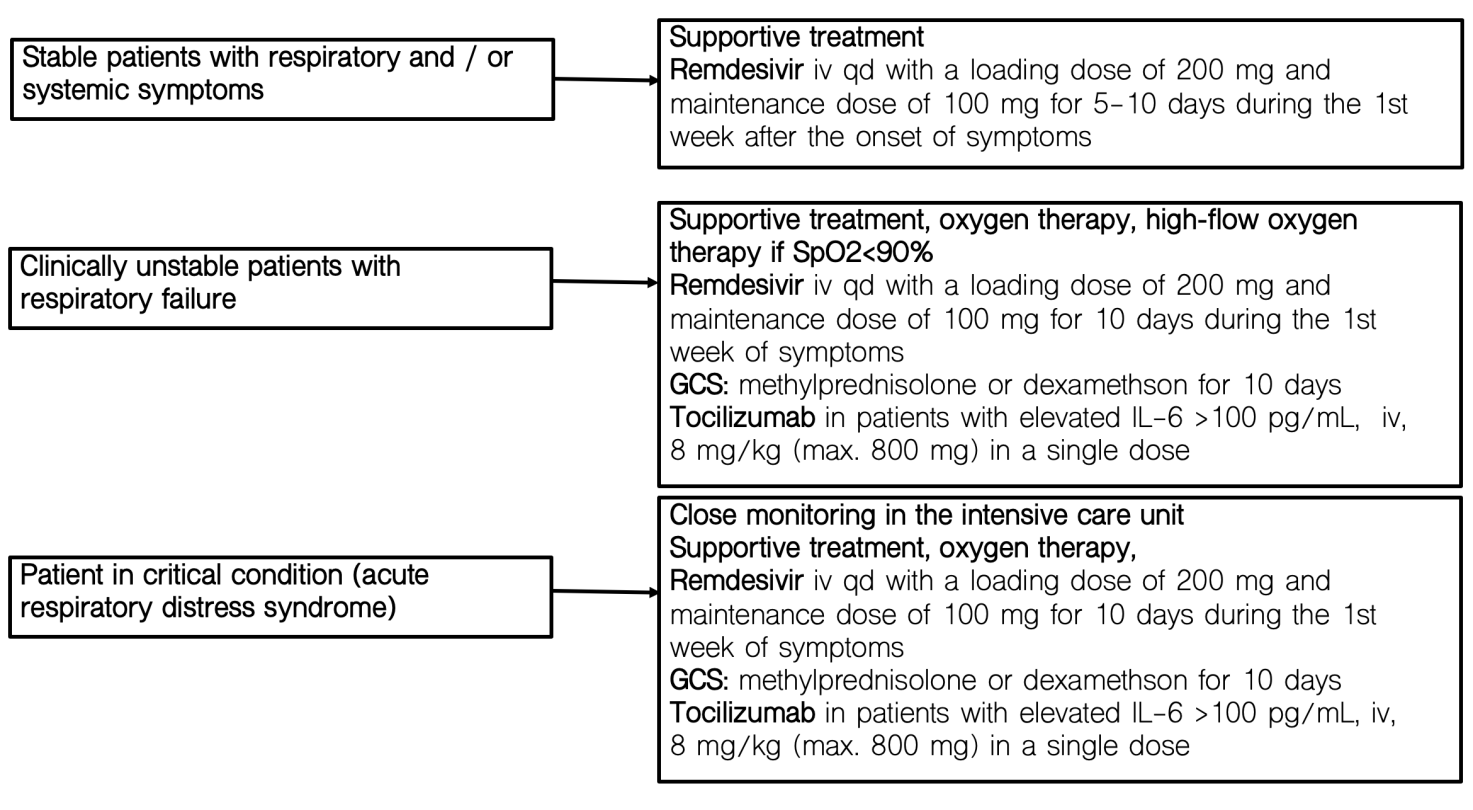
Therapeutic recommendations for COVID-19 at the time of the study.

Patients with oxygen saturation (SpO_2_) of 90% or lower on room air who required high-flow oxygen therapy or non-invasive mechanical ventilation with bilevel positive airway pressure (BiPAP) mode to correct hypoxemia, were categorized as having severe COVID-19. Patients who did not meet the criteria for severe COVID-19 were classified as moderate. Schematic diagram of the treatment regimen is presented in **Figure 1** and **Supplementary Figure 1**.

### 2.2 Sample collection and the purification of peripheral blood mononuclear cells (PBMCs)

Blood samples were collected from each participant by venipuncture in a 7,5 ml S-Monovette tube with clot activator (Sarstedt, Germany) and 4,9 ml S-Monovette tube with EDTA (Sarstedt, Germany) on hospital admission before treatment administration and on the fifth day of treatment. The collected samples were centrifuged, and serum and plasma were frozen at -80 degrees Celsius. Density gradient centrifugation with Pancoll 1.077 g/l (PAN Biotech, Germany) was used for isolation of PBMCs, then the cells were washed twice in phosphatate-buffered saline (Corning, VA, USA) and cryopreserved in fetal bovine serum (PAN Biotech, Germany) with 10% dimethyl sulfoxide (Sigma-Aldrich, MO, USA).

### 2.3 Immunoenzymatic assay (ELISA)

Enzyme-linked immunoabsorbent assays for the quantitative detection of human IL-17A, IL-17F, IL-22 and IL-23 (Invitrogen, Thermo Fisher Scientific, MA, USA) were performed according to the manufacturer’s protocols and an absorbance level was read at 450 nm wavelength using BioTek EL800 microplate reader (Agilent Technologies, CA, USA). The level of each biomarker is expressed in pg/mL.

### 2.4 Multiplex assay

A 17-plex Luminex assay (R&D Systems, MN, USA) was used for quantification of serum biomarkers, including: B7-H3, CCL2, CCL5, CXCL10, IFN-alpha, IFN-gamma, IL-1 beta, IL-4, IL-6, IL-6R, IL-10, IL-13, IL-21, IL-28B, IL-33, GM-CSF, TIM-1. Measurements were performed according to the manufacturer’s instruction on a Bio-Plex 200 System (Bio-Rad Laboratories, CA, USA). The level of each biomarker is expressed in pg/mL.

### 2.5 Flow cytometry

Thawing of cryopreserved PBMCs and resuspension in the complete culture medium (RPMI 1640 with 10% fetal bovine serum (PAN Biotech, Germany)) was followed by counting and viability verification using 0.4% trypan blue solution (cells demonstrated viability of 95-100%). For the flow cytometric assessment of Th17 and Th22 cells, 500,000 cells were used. Intracellular detection of IL-17A and IL-22 was facilitated by 4-hour incubation at 37°C with Leukocyte Activation Cocktail with brefeldin A (BD Pharmingen; BD Bioscience, CA, USA). Initial extracellular staining with monoclonal antibodies conjugated with fluorochromes included: anti-CD4 FITC (clone RPA-T4) and anti-CD8 PE-Cy7 (clone RPA-T8) (BD Bioscience, CA, USA). Following 25 minutes of incubation at room temperature, in the dark, unbound antibodies were washed out with the phosphate-buffered saline without calcium and magnesium (PBS; Corning, VA, USA). Subsequently, cells were permeabilized by Permeabilization Buffer 2 (BD Bioscience, CA, USA) for intracellular staining using: anti-IL-17A PE (clone SCPL1362) and anti-IL-22 Alexa Fluor 647 (clone MH22B2) (BD Bioscience, CA, USA). After incubation and washing steps, cells were preserved using CellFix reagent (BD Bioscience, CA, USA) and stored at 4°C prior to data acquisition on the FACS Calibur flow cytometer (BD Bioscience, CA, USA). Flow cytometric analyses were performed using the FlowJo^®^ software (Tree Star Inc., OR, USA).

**Figure 2:**
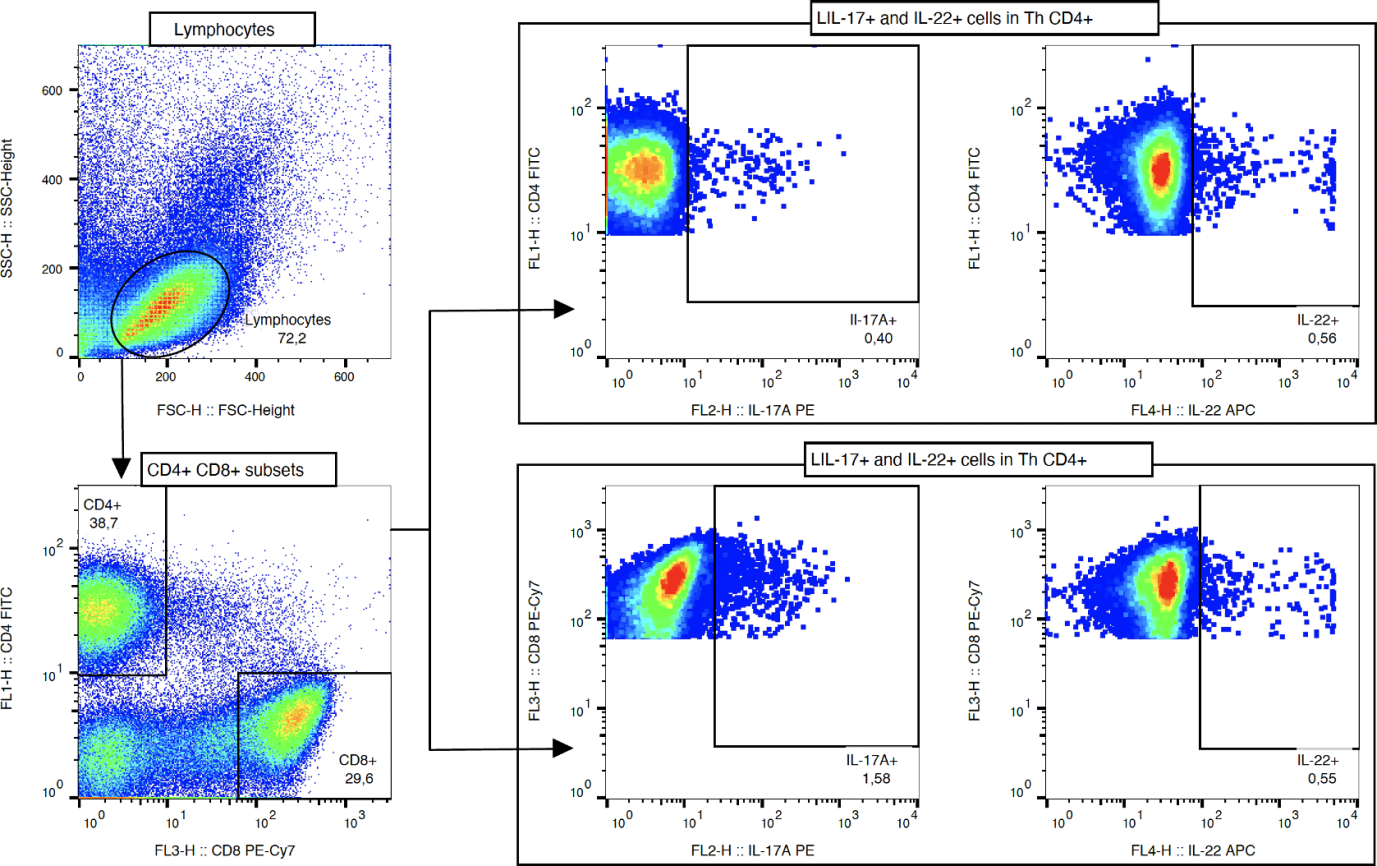
The representative gating strategy, based on proper FMO and unstained controls.

Lymphocytes were initially distinguished on the basis of their morphology: relative size (FSC; forward scatter) and granularity (SSC; side scatter). Next, the detection of the helper (Th) CD4+ and cytotoxic (Tc) CD8+ T cell subsets was performed. Furthermore, the intracellular expression of IL-17A and IL-22 was assessed within mentioned subsets of lymphocytes. Implemented processing of the flow cytometric data allowed for determining the studied T cell populations frequencies and absolute cell numbers. The representative gating strategy, based on proper FMO and unstained controls, is presented in **Figure 2**.

### 2.6 Statistical analysis

Statistical analysis was performed with GraphPad (Prism Software, CA, USA). Comparative analysis between groups according to the distribution was carried out by t-test or Mann-Whitney U test for continuous data and chi-square for categorical data. For comparison between groups before and during the treatment, paired t-test and Wilcoxon matched pairs signed rank test were applied followed by two-stage linear step-up procedure of Benjamini, Krieger and Yekutieli. To assess the panoramic profile of serum biomarkers and absolute counts of immune cells in peripheral blood, data was normalized with log transformation. The correlation between analyzed parameters was assessed with Spearman correlation test. The differences were considered statistically significant if p<0.05.

## 3 Results

### 3.1 General characteristics

**Figure 3:**
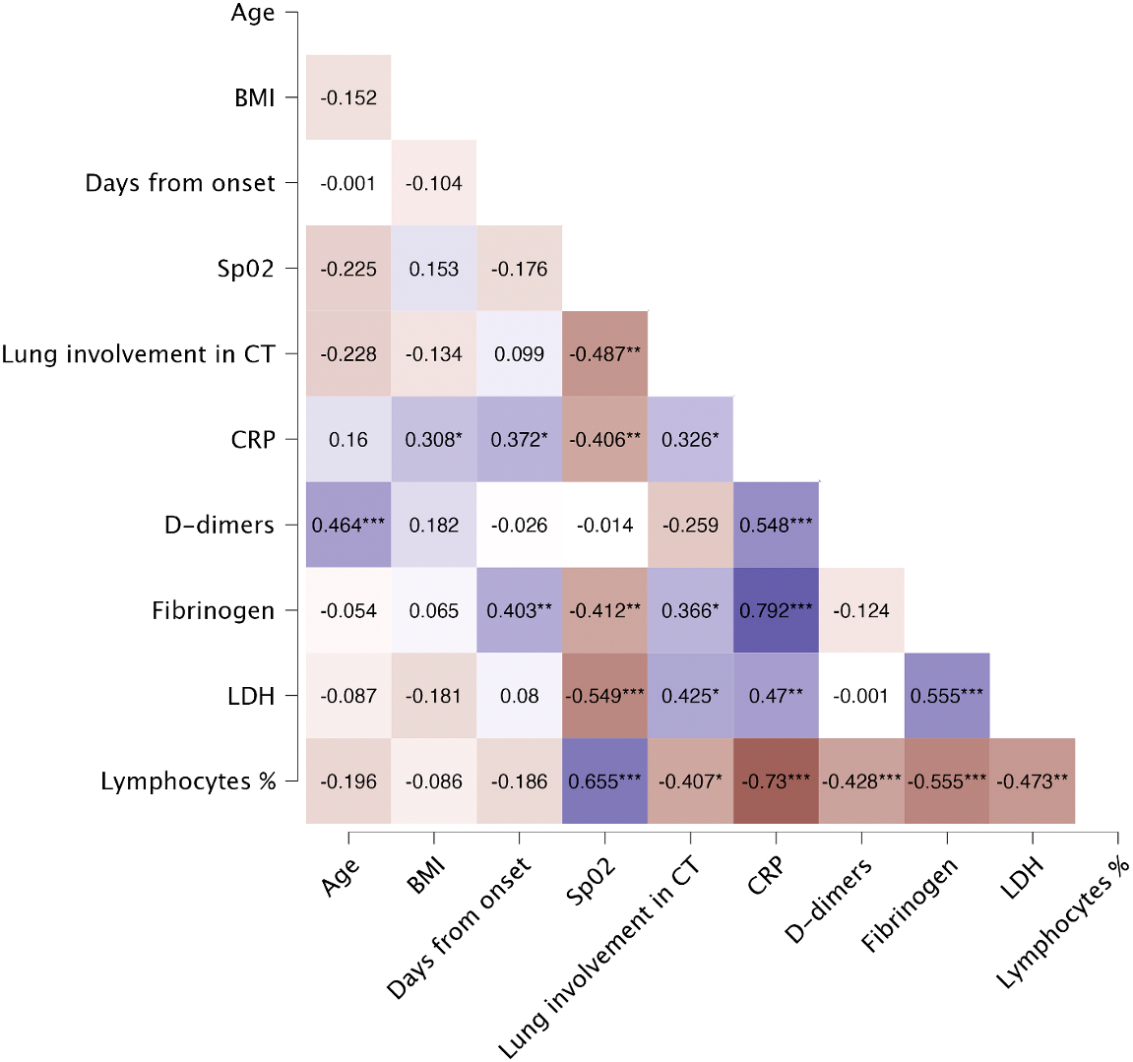
Spearman’s correlation of laboratory findings and general characteristics of patients on the day of hospital admission. Heatmap demonstrates exact values of Spearman’s r. BMI, body mass index; CRP, C-reactive protein; CT, computed tomography; LDH, lactate dehydrogenase; Lymphocytes %, percentage of lymphocytes; SpO_2_, oxygen saturation. The levels of significance were indicated as: * = p<.05, ** = p<.01, *** = p <.001.

General characteristics of patients (n=44) and controls (n=20) are presented in **Table 1**. Among patients, majority were male (72.7%) with a median age of 63 years, whereas among controls majority were female (55.0%) with a median age of 57. Among severe COVID-19, all patients were male with a median age of 67 years (**Supplementary Table 1**). The most frequently occurring comorbidities both in patients and controls were hypertension (56.8% and 20.0% respectively) and dyslipidaemia (18.2% and 10.0% respectively). The median time from onset of symptoms to hospitalization was 7.0 days. On hospital admission, the median of the lung involvement in CT scan was 40.0%, whereas the median of oxygen saturation was 90.0%. Lung involvement in CT scan showed significant negative correlation with oxygen saturation (r=-0.487, p=0.002) and lymphocytes percentage (r=-0.407, p=0.010), and positive correlation with LDH level (r=0.425, p=0.011). Heatmap of Spearman’s correlation of laboratory findings and general characteristics is presented in **Figure 3**.

**Table 1:** General characteristics of COVID-19 patients and control group.

Variables	Patients (n=44)	Healthy controls (n=20)	p
Age, y	63.0 (51.5-70.5)	57.0 (51.5-63.0)	0.087
Gender, male	32 (72.7%)	9 (45.0%)	0.032*
BMI, kg/m^2^	29.7 (27.7-33.9)	26.0 (24.2-30.7)	0.010*
SpO_2_, %	90.0 (84.5-94.0)	–	–
Time from onset, days	7.0 (5.0-9.0)	–	–
Disease severity			
Moderate, n (%) Severe, n (%)	35 (79.5%) 9 (20.5%)	- -	- -
Comorbidities			
Hypertension, n (%) Diabetes, n (%) Asthma, n (%) Dyslipidaemia, n (%)	25 (56.8%) 7 (16.0%) 3 (6.8%) 8 (18.2%)	4 (20.0%) 0 (0.0%) 1 (5.0%) 2 (10.0%)	0.010* 0.059 0.937 0.403
Symptoms			
Fever, n (%) Cough, n (%) Dyspnoea, n (%) Fatigue, n (%)	32 (72.7%) 28 (63.6%) 24 (54.6%) 23 (52.3%)	- - - -	–
Lung involvement in CT scan on admission, %	40.0 (40.0-60.0)	–	–

Data represented as number or median (IQR). BMI, body mass index; SpO_2_, oxygen saturation; *, statistical significance.

### 3.2 Laboratory findings

On admission 65.9% of patients showed lymphocytopenia, while leukocyte counts were normal. Alanine aminotransferase, aspartate aminotransferase and D-dimer levels were significantly higher in patients than in controls (p=0.006, p<.001 and p<.001 respectively). Detailed analysis of laboratory findings in patients and controls is presented in **Table 2**. LDH levels were significantly higher in patients with severe COVID-19, compared to patients with moderate disease (p=0.040) (**Supplementary Table 2**). Lymphocytes percentage correlated negatively with CRP (r=-0.730, p<.001), fibrinogen (r=-0.555, p<.001), LDH (r=-0.473, p<.001), and D-dimers (r=-0.428, p<.001). Additionally, lymphocytes percentage showed strong positive correlation with SpO_2_ (r=0.655, p<.001).

**Table 2:** Laboratory findings in COVID-19 patients and control group.

Variables	Normal range	Patients (n=44)	Healthy controls (n=20)	p
ALT, IU/L	<31.0	46.0 (23.0-62.0)	24.5 (20.0-30.0)	0.006*
AST, IU/L	<32.0	53.0 (44.0-74.0)	22.0 (19.0-29.0)	<.001*
CRP, mg/dL	<5.0	93.6 (58.9-132.3)	1.1 (0.6-2.4)	<.001*
PCT, ng/mL	<0.05	0.09 (0.05-0.16)	–	–
D-dimers, ng/mL	<500.0	892.0 (638.0-1439.0)	250.0 (182.0-382.0)	<.001*
Fibrinogen, mg/dL	200.0-400.0	645.0 (527.0-758.0)	–	–
LDH, U/I	135.0-214.0	476.0 (345.3-615.5)	–	–
Leukocyte count, x10^9^/L	4.0-10.0	5.6 (4.7-8.7)	5.8 (5.0-7.3)	0.902
Neutrophil count, x10^9^/L	1.6-7.2	4.3 (3.1-7.3)	3.1 (2.5-4.1)	0.018*
Lymphocyte count, x10^9^/L	0.8-4.7	0.8 (0.7-1.2)	2.0 (1.6-2.5)	<.001*
Lymphocytes, %	18.0-48.0	12.8 (8.8-20.1)	32.4 (28.4-36.9)	<.001*
Haemoglobin, g/dL	12.0-16.0	14.8 (13.3-15.4)	14.4 (13.6-15.3)	0.848
Platelet count, x10^9^/L	130.0-350.0	183.0 (139.0-261.5)	233.0 (207.0-294.0)	0.015*
INR	0.8-1.2	1.2 (1.1-1.2)	1.0 (1.0-1.1)	<.001*

Data represented as number or median (IQR). ALT, alanine aminotransferase; AST, aspartate aminotransferase; CRP, C-reactive protein; INR, international normalized ratio; LDH, lactate dehydrogenase; PCT, procalcitonin; *, statistical significance.

### 3.3 Immunological features before the treatment

**Figure 4:**
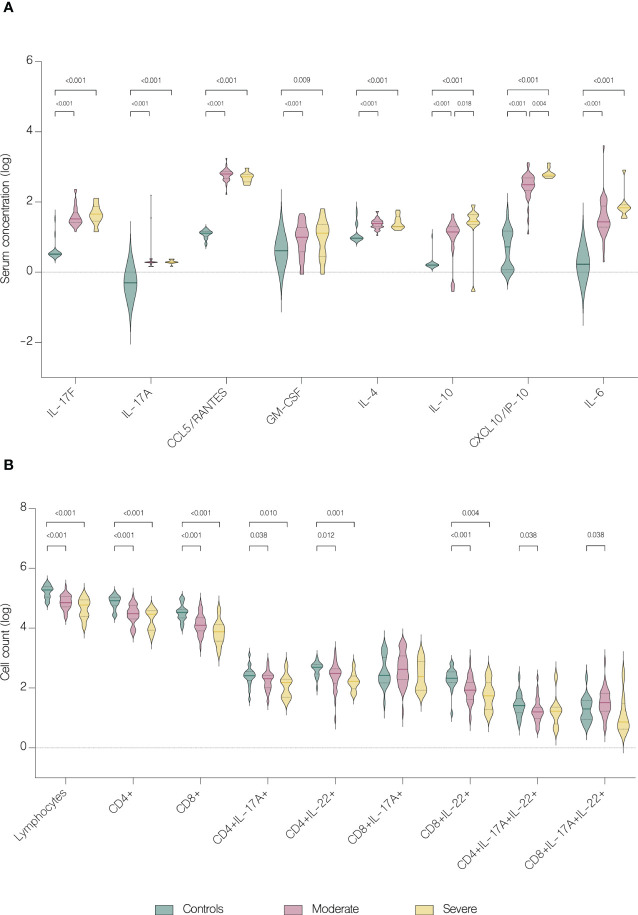
Immunological features in healthy controls and patients. Data represented as median (—) and quartiles (^……^). **(A)** Serum concentration of cytokines in healthy controls and patients with moderate and severe disease on hospital admission; **(B)** T cell counts in healthy controls and patients with moderate and severe disease on hospital admission.

**Figure 5:**
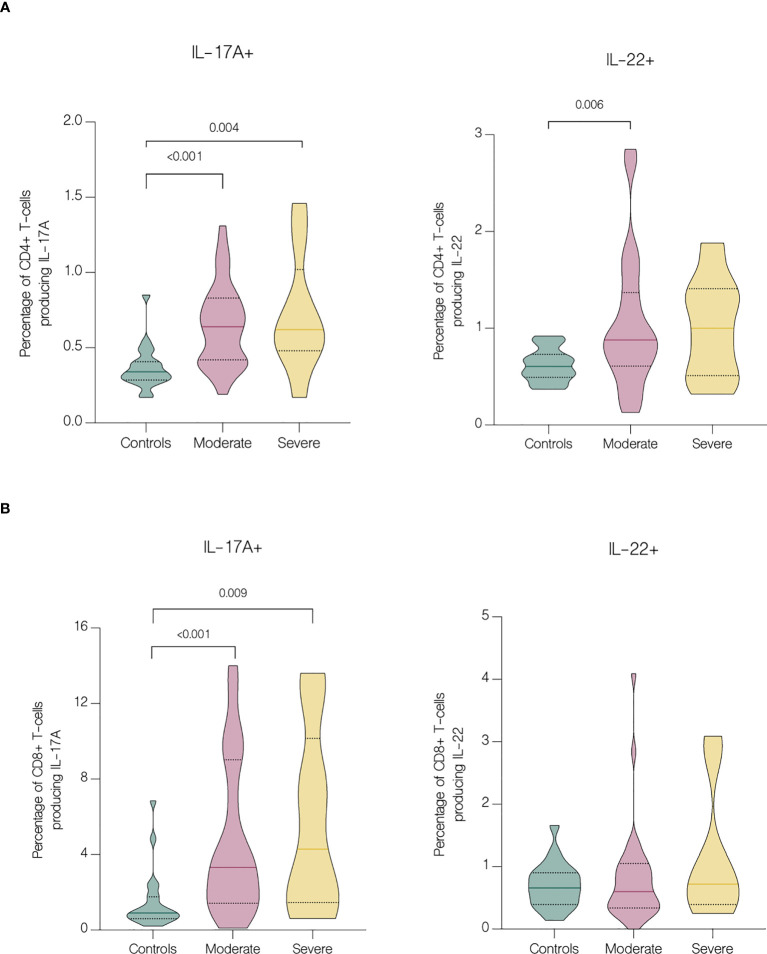
Intracellular expression of IL-17A and IL-22 by CD4+ and CD8+ T cells in controls and patients with moderate and severe disease. Data represented as median (—) and quartiles (^……^). **(A)** Cytokine production by CD4+ cells in controls and patients with moderate and severe disease on hospital admission; **(B)** Cytokine production by CD8+ cells in controls and patients with moderate and severe disease on hospital admission.

**Figure 6:**
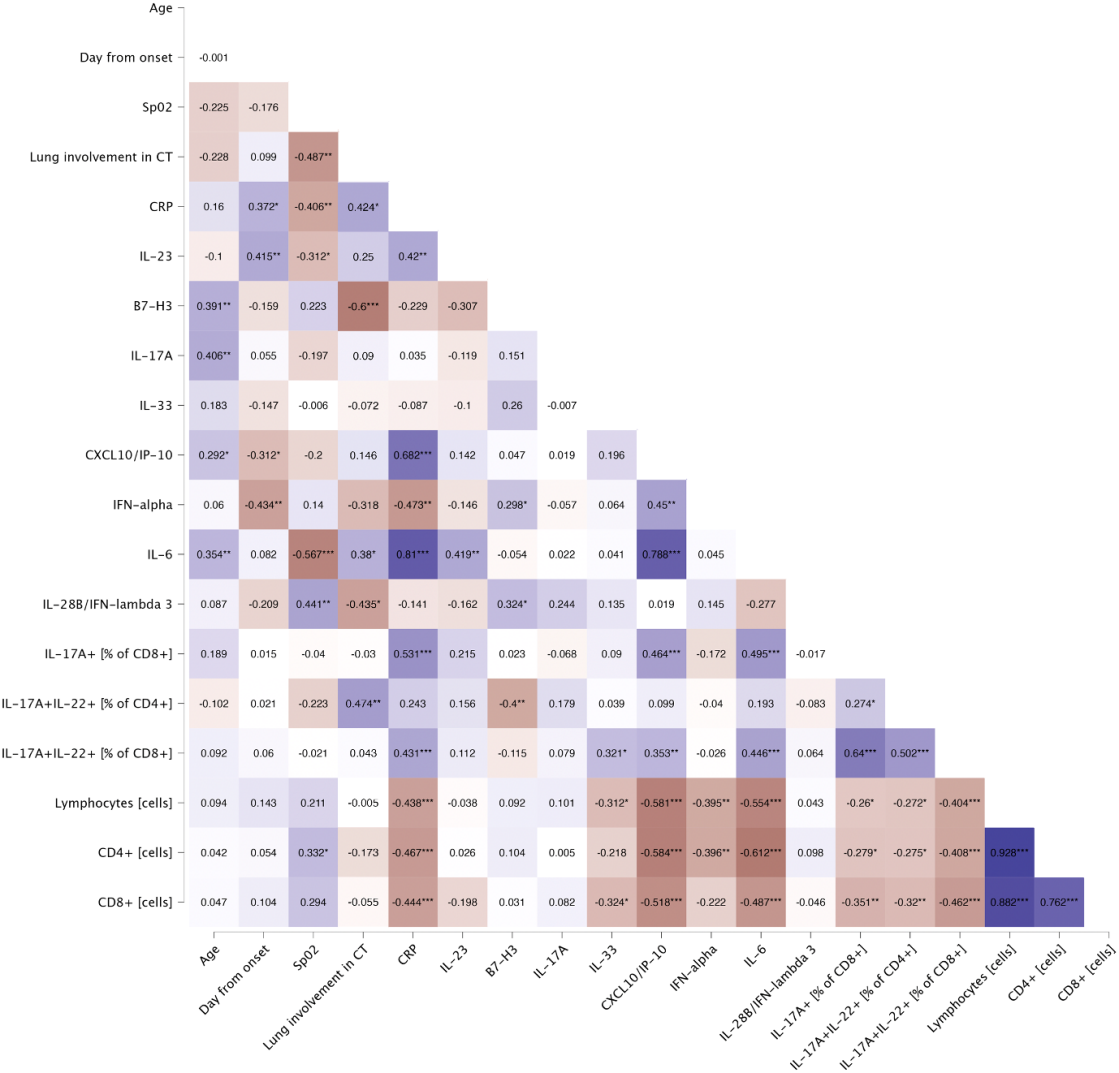
Spearman’s correlation of general characteristics and immunological features of patients on the day of hospital admission. Heatmap demonstrates exact values of Spearman’s r. CRP, C-reactive protein; CT, computed tomography; SpO_2_, oxygen saturation. The levels of significance were indicated as: * = p<.05, ** = p<.01, *** = p <.001.

Serum concentrations of IL-17F (p<.001), IL-17A (p=0.018), CCL5/RANTES (p<.001), GM-CSF (p<.001), IL-4 (p<.001), IL-10 (p<.001), CXCL10/IP-10 (p<.001) and IL-6 (p<.001) were significantly upregulated in patients, comparing to healthy controls (**Figure 4A**). Furthermore, IL-10 and CXCL10/IP-10 were significantly higher in patients with severe COVID-19 (p=0.018 and p=0.004 respectively) (**Figure 4A**). Whereas analysis of circulating immune cell subsets demonstrated significant decrease of absolute numbers of total lymphocytes, as well as both CD4+ and CD8+ T cells (**Figure 4B**). Nevertheless, both the percentages of CD4+ and CD8+ T cells expressing IL-17A were higher in patients with moderate (p<.001 and p<.001 respectively) and severe disease (p=0.004 and p=0.009 respectively) than in controls. In addition, patients with moderate disease had higher percentage of CD4+ T cells expressing IL-22 (p=0.006) compared to controls (**Figure 5**). Lung involvement in CT scan correlated positively with percentage of CD4+ T cells producing both IL-17A and IL-22 (r=0.474, p=0.005) and negatively with IFN-lambda 3 (r=-0.435, p=0.010). In contrast, SpO2 correlated positively with IFN-lambda 3 (r=0.441, p=0.007) and negatively with IL-6 (r=-0.567, p=0.007). CRP correlated positively with percentage of CD8+ producing IL-17A (r=0.531, p<.001) and percentage of CD8+ producing both IL-17A and IL-22 (r=0.431, p<.001). Moreover, CRP correlated negatively with lymphocytes count (r=-0.438, p<.001) as well as CD4+ (r=-0.467, p<.001) and CD8+ (r=-0.444, p<.001). Time from onset correlated positively with IL-23 (r=0.415, p=0.009) (**Figure 6**).

### 3.4 Effect of treatment on immunological features of patients

**Figure 7:**
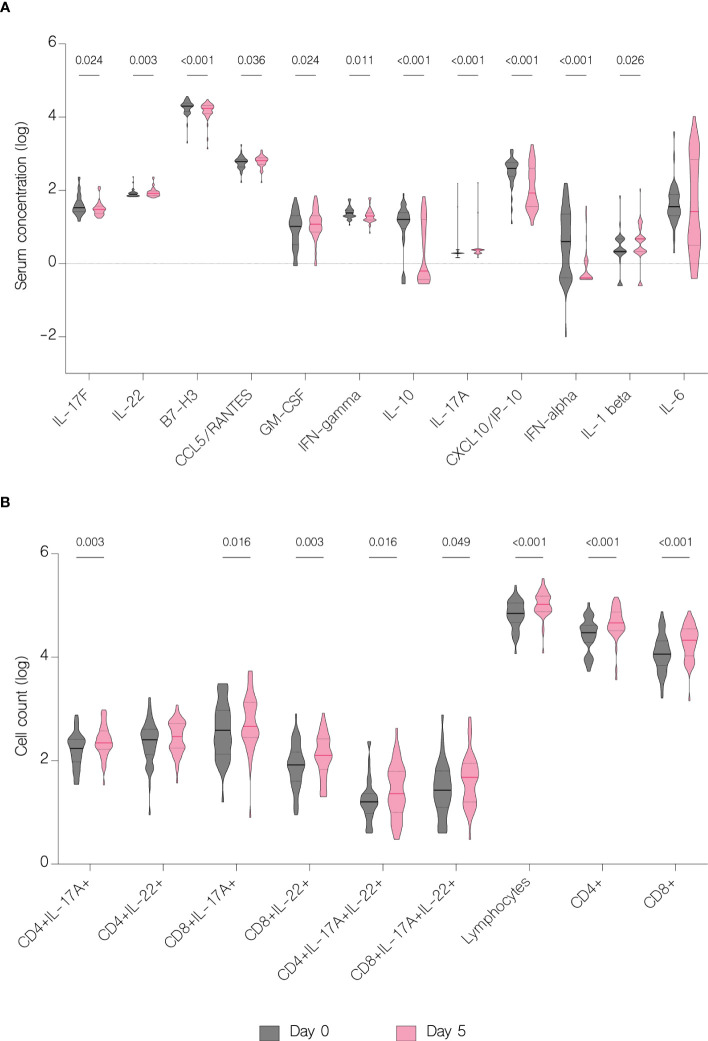
Immunological features before and during treatment. Data represented as median (—) and quartiles (^……^). **(A)** Serum concentration of cytokines before treatment administration and on the fifth day of treatment; **(B)** T cell counts before treatment administration and on the fifth day of treatment.

**Figure 8:**
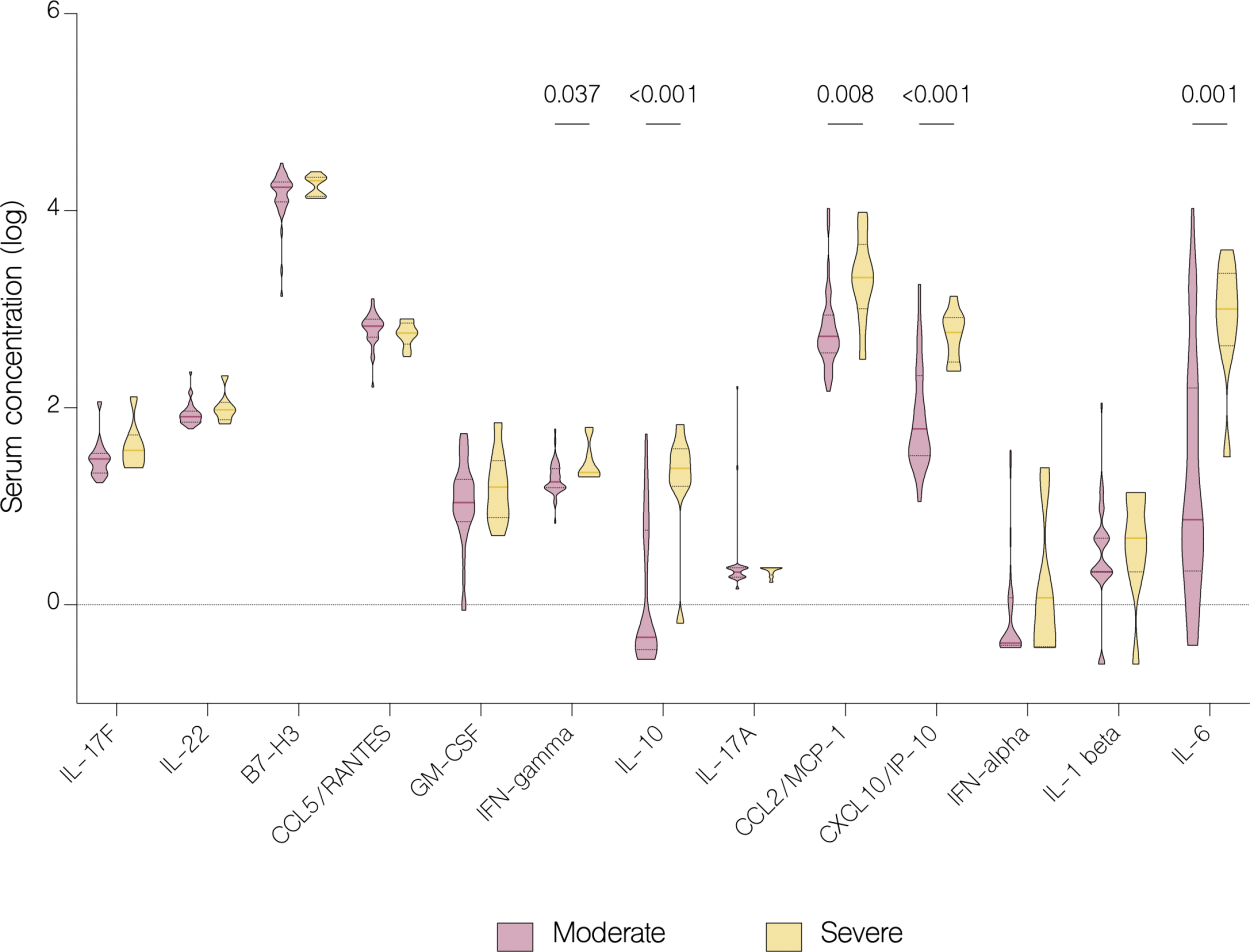
Serum concentration of cytokines in patients with moderate and severe COVID-19 on the fifth day of treatment. Data represented as median (—) and quartiles (^……^).

On the fifth day of treatment, the overall level of IL-17A (p<.001), IL-22 (p=0.003), IL-1 beta (p=0.026), CCL5/RANTES (p=0.036) and GM-CSF (p=0.024) significantly increased. Whereas the level of IL-17F (p=0.024), IL-10 (p<.001), IFN-gamma (p=0.011), IFN-alpha (p<.001), CXCL10/IP-10 (p<.001) and B7-H3 (<.001) significantly decreased (**Figure 7A**). However, compared to patients with moderate COVID-19, the overall serum levels of IL-10 (p<.001), IFN-gamma (p=0.037), CCL2/MCP-1 (p=0.008), CXCL10/IP-10 (p<.001) and IL-6 (p=0.001) were significantly upregulated in severe patients on fifth day of treatment (**Figure 8**).

**Figure 9:**
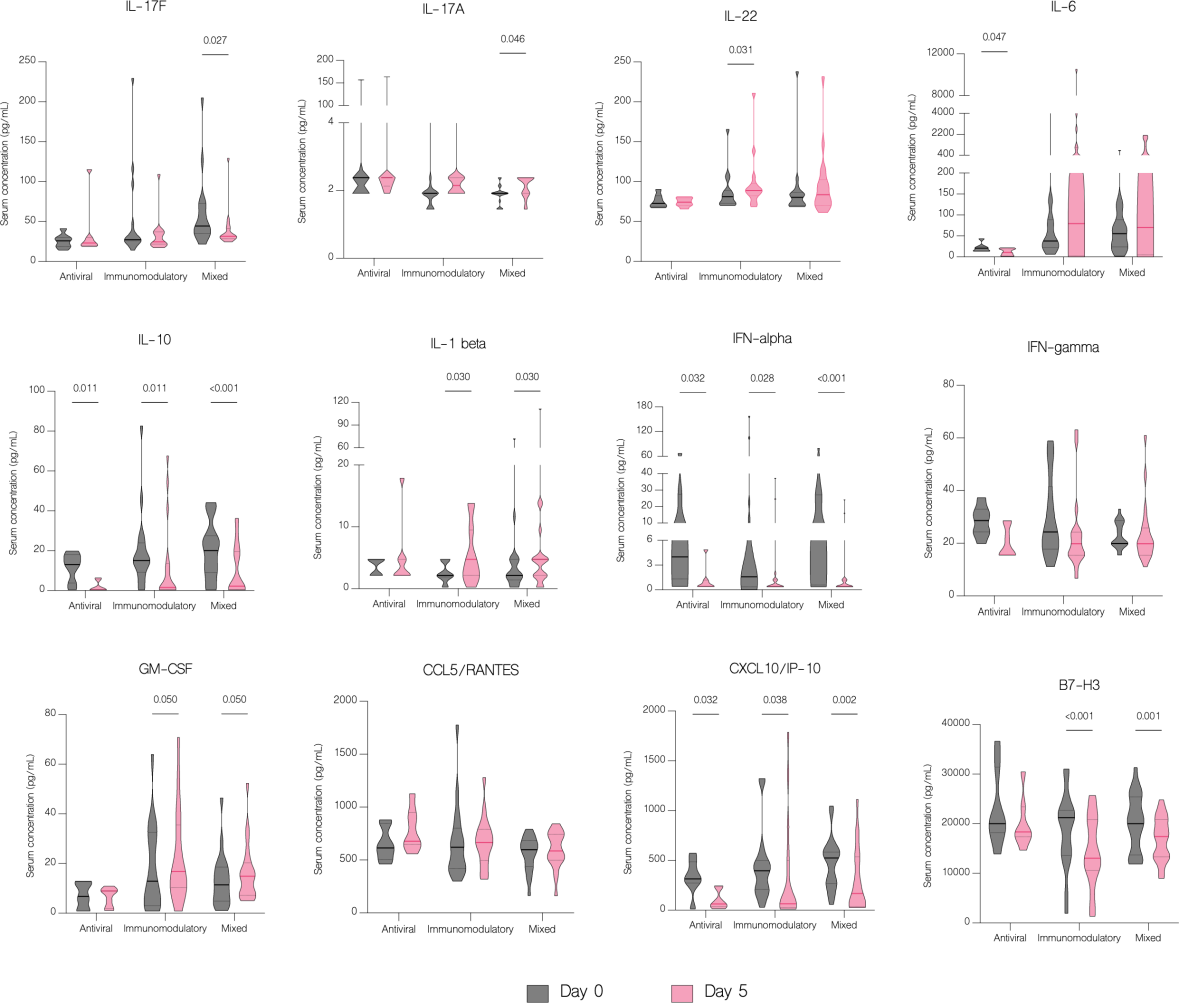
The effect of treatment on a cytokine profile in different treatment groups. Serum concentration of cytokines in different treatment groups, classified as follows: antiviral – remdesivir; immunomodulatory – tocilizumab or/and dexamethasone; mixed – combination of antiviral and immunomodulatory treatment. Data represented as median (—) and quartiles (^……^).

**Figure 10:**
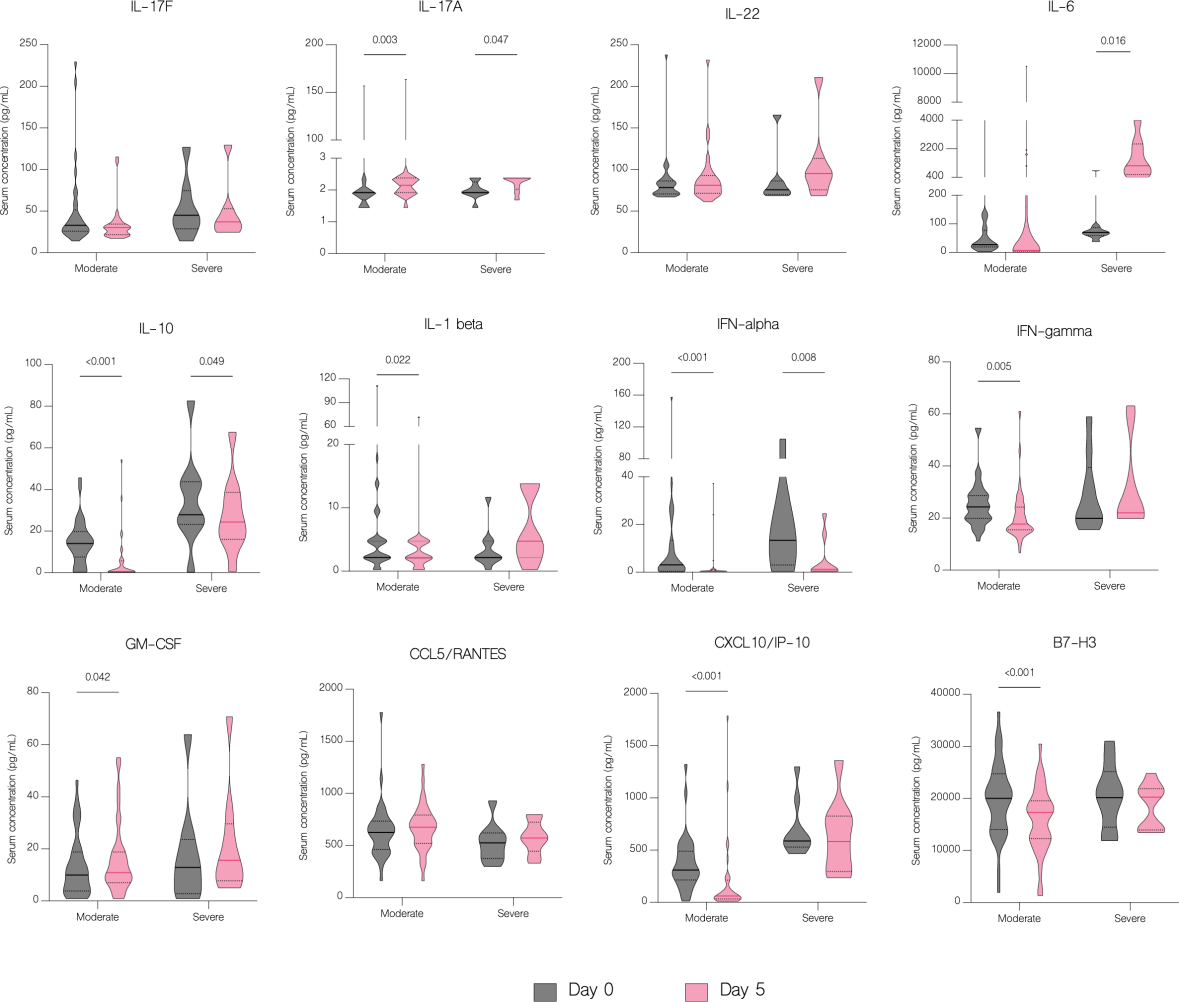
The effect of treatment on a cytokine profile by severity. Serum concentration of cytokines in patients with moderate and severe disease before treatment administration and on the fifth day of treatment. Data represented as median (—) and quartiles (^……^).

IL-6 concentration decreased significantly with antiviral treatment (p=0.047) but increased in patients in other treatment groups, although without statistical significance (p=0.067) (**Figure 9**). Moreover, there was a statistically significant increase of IL-6 in patients with severe COVID-19 (p=0.016) compared to patients with moderate disease (**Figure 10**). Compared to other therapies, only patients with immunomodulatory treatment showed an increase of IL-22 level (p=0.031), whereas combination of immunomodulatory and antiviral treatments resulted in a significant increase of IL-17A (p=0.046) and a decrease of IL-17F (p=0.027) in these patients (**Figure 9**). Concentration of IL-1 beta increased significantly in both groups treated with immunomodulatory drugs (p=0.030) and a combination of immunomodulatory and antiviral drugs (p=0.030) (**Figure 9**). In contrast to the group with severe COVID-19, in patients with moderate disease IL-1 beta levels were significantly reduced (p=0.022) on fifth day of treatment, suggesting its contribution to worse clinical outcomes (**Figure 10**).

**Figure 11:**
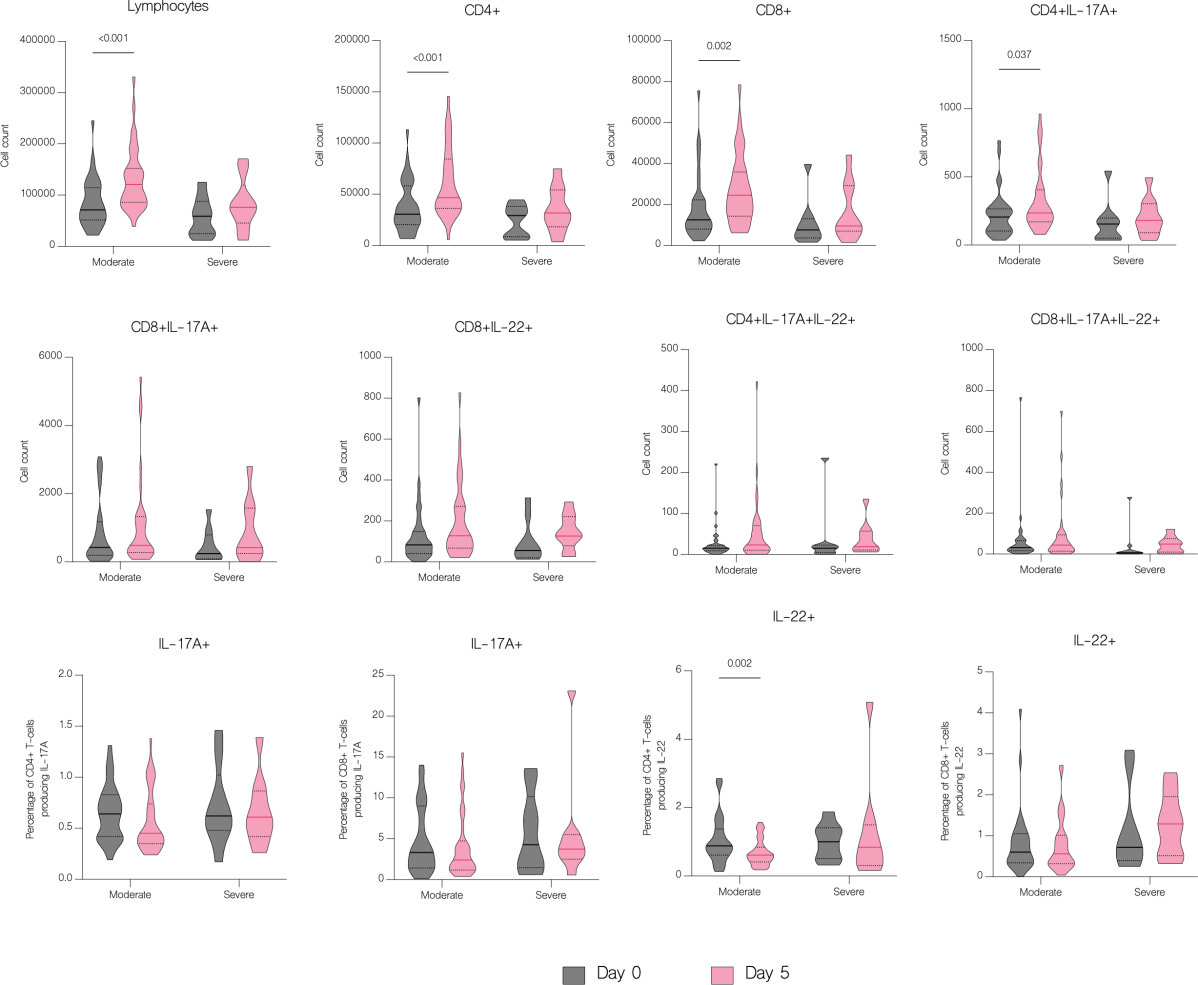
The effect of treatment on T cell counts in patients by severity. T cell counts before treatment administration and on the fifth day of treatment in patients with moderate and severe disease. Data represented as median (—) and quartiles (^……^).

**Figure 12:**
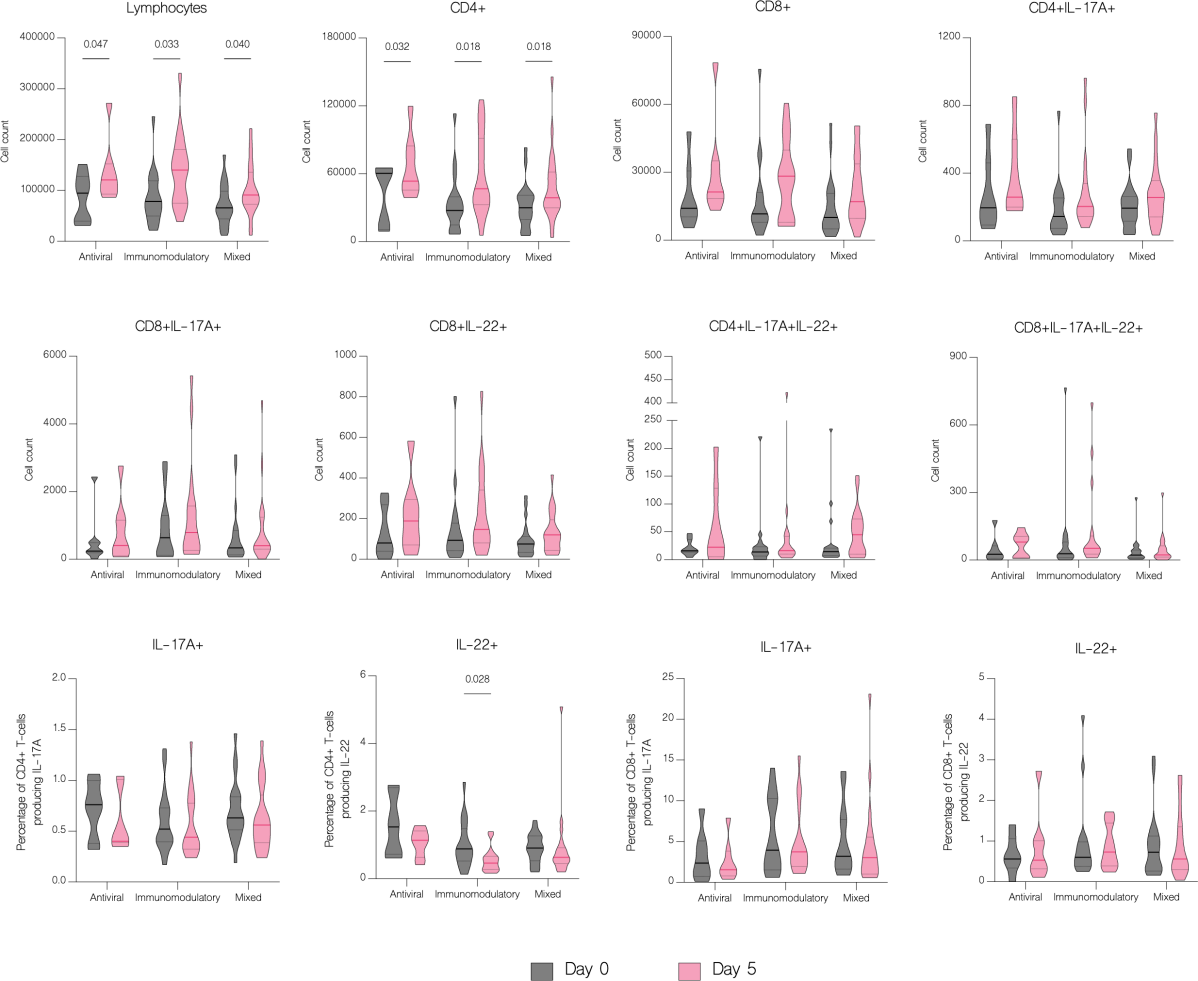
The effect of treatment on T cell counts in patients in different treatment groups. T cell counts before treatment administration and on the fifth day of treatment in different treatment groups, classified as follows: antiviral – remdesivir; immunomodulatory – tocilizumab or/and dexamethasone; mixed – combination of antiviral and immunomodulatory treatment. Data represented as median (—) and quartiles (^……^).

The overall number of lymphocytes, and both CD4+ and CD8+ T cell subsets significantly increased with treatment (p<.001), including CD4+ and CD8+ producing IL-17A (p=0.003 and p=0.016 respectively) (**Figure 7B**). However, patients with moderate disease demonstrated immune response to treatment with a significant increase in both CD4+ and CD8+ T cell counts (p<.001 and p=0.002 respectively), while in patients with severe disease there was no significant difference in any subset (p=0.126 and p=0.152 respectively) (**Figure 11**). There were no statistically significant differences of T cell counts between the different treatment groups, however the percentage of IL-22 producing CD4+ T cells was markedly lower in patients treated with immunomodulatory drugs (p=0.035) (**Figure 12**).

## 4 Discussion

Laboratory parameters have potential value in risk assessment and COVID-19 outcomes prediction, as early recognition of disease progression is essential for effective management and intervention. Several biomarkers were proposed as potential predictors of the COVID-19 outcome based on the hyperinflammatory state and hypercoagulability involved in pathophysiology of severe disease, such as CRP, PCT, IL-6, D-dimers and LDH (14). Many studies reported that elevated levels of ALT, AST, CRP, PCT, IL-6, D-dimers and LDH, and depressed counts of lymphocytes and platelets in patients with COVID-19 were associated with disease severity (14–19). Consistently with previous findings, in our study patients had significantly higher levels of ALT, AST, CRP and D-dimers and lower counts of lymphocytes and platelets compared to controls. In addition, patients had elevated levels of PCT, fibrinogen and LDH. Moreover, patients with severe disease had significantly higher level of LDH, while there was no statistical difference in other laboratory parameters.

Impairment of immune responses resulting in an excessive inflammation is a hallmark of COVID-19. In addition to the virus-induced direct lung injury, excessive activation of immune system in response to SARS-CoV-2 invasion triggers immune cells to release both pro- and anti-inflammatory cytokines, such as IL-1 beta, IL-6, IL-10, IL-17, TNF and CCL2/MCP-1 (20). We have demonstrated that concentrations of several cytokines, including CCL5/RANTES, GM-CSF, IL-4, IL-6, IL-10 and CXCL10/IP-10, were significantly upregulated in patients with COVID-19 versus healthy controls. Moreover, upon admission patients with severe disease had higher concentration of IL-10 and CXCL10/IP-10 than patients with moderate disease. These results are in accordance with the studies reporting an elevation of IL-1 beta, IL-6, IL-10, GM-CSF, CXCL10/IP-10, CCL2/MCP-1, CCL5/RANTES and IFN-gamma in the blood samples of COVID-19 patients (21–23). Furthermore, Huang et al. observed higher concentration of IL-10, CXCL10/IP-10 and CCL2/MCP-1 in intensive care COVID-19 patients versus non intensive care patients (21).

Several studies reported decrease in CD4+ and CD8+ T cells counts in patients with COVID-19 (23–27). Hence, it has been suggested that the counts of CD4+ and CD8+ T cells can be a diagnostic marker of COVID-19 activity and predictor of disease severity (25). Patients in our study had significantly reduced both CD4+ and CD8+ absolute counts, but without significance between moderate and severe disease. However, on fifth day of hospitalization patients with severe disease had lower lymphocytes, CD4+ and CD8+ T cells count, which may be explained by the fact that in majority of severe cases, the disease progressed during hospitalization. In contrast to the group with severe COVID-19, patients with moderate disease showed clinical response to treatment, with a significant increase in both CD4+ and CD8+ T cells count. Similar results were obtained by Wang et al. showing an increase in CD8+ T cells and B-cells in response to treatment in patients with COVID-19. Moreover, authors indicated that the decrease of these cell subsets might serve as an independent predictor of poor treatment response (26). In our study, despite the depleted absolute numbers of CD4+ and CD8+ T cells, these cells showed overactivation and increased expression of IL-17A and IL-22. We found significantly higher percentage of both CD4+ and CD8+ T cells producing IL-17A and CD4+ T cells producing IL-22 in patients with COVID-19. These results correspond to the study of De Biasi et al. that reported an increase in percentages of T cell subsets producing IL-17A (22). Similarly, Cagan et al. reported increased numbers of CD4+ and CD8+ T cells producing IL-22 and CD8+ T cells producing IL-17A (7).

The production of IL-17A, a cytokine with both protective and pathogenic role, is the hallmark of the Th17 response. IL-17A has the ability to activate a wide range of inflammatory pathways, which can cause tissue damage and illness aggravation. Several indicators pointed to the likely role of IL-17A in COVID-19 clinical outcomes prompting considerations of using this cytokine as a marker of disease severity (20, 28–30). Previous study noted significantly upregulated IL-17A and IL-17F in patients with COVID-19 (23). Likewise, patients with COVID-19 in our study had significantly greater level of IL-17A and IL-17F than controls, but without significance between patients with moderate and severe disease. This observation is in accordance with the study of Huang et al. that reported significantly higher concentration of IL-17A in ICU patients compared to healthy controls but without significance between ICU and non-ICU care (21). Similarly, Mostafa et al. also noted upregulated levels of IL-17A in paediatric patients with COVID-19, however no association with disease outcome was found (31). In contrast, Liu et al. demonstrated significantly higher level of IL-17 in patients with severe COVID-19 compared to patients with mild disease. Moreover, IL-17 has been shown to positively correlate with Murray score, an indicator of lung injury severity (32). Another study showed an increase of IL-17 both in plasma and saliva of patients with severe COVID-19 compared to mild and asymptomatic patients. In addition, elevated levels of IL-17 were reported in nasal swabs and lung autopsies of COVID-19 patients and were associated with higher concentrations of other proinflammatory cytokines, including IL-1 beta, IL-6, IL-8, and IL-23 (33).

Another cytokine associated with Th17 response with both protective and pathogenic role is IL-22. It regulates host defense at barrier surfaces and promotes tissue regeneration yet has been linked to several diseases characterized by inflammatory tissue pathology (34). However, recent study of Das et al. reported potent immune boosting and antiviral properties of IL-22 in respiratory syncytial virus (RSV) infection and since COVID-19 have pathological characteristics like other viral respiratory infections it is reasonable to speculate that IL-22 may also limit the severity of SARS-CoV-2 infection (34, 35). We found elevated levels of IL-22 in patients on admission. In addition, concentrations of IL-22 in our patients significantly increased during hospitalization. Likewise, previous studies showed upregulated levels of IL-22 in both paediatric and adult patients with COVID-19, however no association with disease outcome or severity was found (23, 31). During inflammation, group 3 innate lymphoid cells (ILC3s) produce IL-17 and IL-22 upon stimulation with IL-1 beta and IL-23. In our study, despite no significant increase in T cell subsets in patients with severe disease there was an increase in IL-17A and IL-22 concentration which might be associated with ILC3s. In addition, it has been shown that lower abundance of ILCs in the blood was associated with longer hospitalization in individuals with COVID-19, hence there might be a correlation between decreased ILCs in the blood and severe disease (36, 37).

The number of available studies on the effect of antiviral and immunomodulatory treatment on a cytokine profile in patients with COVID-19 is scarce, hence the impact of treatment on the immune response is still unclear. In our study, treatment with remdesivir (RDV) resulted in a significant decline in concentrations of IL-6, IL-10, IFN-alpha and CXCL10/IP-10. Moreover, IFN-gamma and IL-1 beta were markedly decreased in patients treated with RDV, although without statistical significance. We were unable to find another study to which to compare our findings, however our results suggest attenuation of an excessive immune response in patients receiving antiviral treatment. Immunomodulatory treatment resulted in a significant downregulation of IL-10, IFN-alpha, CXCL10/IP-10 and B7-H3 as well as upregulation of IL-22 and IL-1 beta. The report of Ponthieux et al. showed an increase in IL-1 beta, IL-2, IL-4, IL-10, IL-12p70, IL-18 and IL-6R levels in patients with COVID-19 treated with tocilizumab achieving maximal values two to four days after drug administration. The authors of the study attributed these findings to an anti-inflammatory effect of IL-6, which was previously observed by other researchers (38, 39). However, in an experimental model of oleic acid-induced acute lung injury (ALI) both tocilizumab (TCZ) and dexamethasone (DEX) significantly reduced the expression of IL-1 beta, IL-6, IL-8, and TNF-alpha (40). Furthermore, DEX significantly reduced IL-8 levels in tracheal aspirates of mechanically ventilated children with RSV infection (41). In addition, DEX inhibited the secretion of IL-6 and CXCL8 in human lung fibroblasts (42). Another study with an oleic acid-induced ALI demonstrated that DEX inhibits the expression of IL-6, TNF-alpha and VEGF while promoting the expression of IL-10 (43). Immunomodulatory treatment inhibits the expression of proinflammatory cytokines and promotes the expression of anti-inflammatory cytokines, hence alleviating tissue damage during infections. In our study, a combination of an antiviral and immunomodulatory treatment resulted in a significant decrease in IL-17F, IL-10, IFN-alpha, CXCL10/IP-10 and B7-H3 levels as well as an increase in IL-17A and IL-1 beta. However, an upregulation of proinflammatory cytokines, such as IL-17A and IL-1 beta, might be associated with higher number of severe cases and deaths in this group, if untreated. In fact, IL-1 beta was significantly lower in patients with moderate disease and markedly higher in patients with severe disease, although without statistical significance. Lastly, patients treated with antiviral drug, immunomodulatory drug or both demonstrated clinical response to treatment with an increase in lymphocyte, CD4+ and CD8+ counts, without difference between the treatment groups.

## 5 Conclusions

SARS-CoV-2 infection induced cytokine overexpression in hospitalized patients with COVID-19 as well as lymphopenia, particularly a decrease in CD4+ and CD8+ T cell counts. Moreover, despite the reduced counts of CD4+ and CD8+ T cells, both subsets showed overactivation and increased expression of IL-17A and IL-22, thus targeting Th17 response might alleviate inflammatory response in severe disease.

Administration of antiviral or/and immunomodulatory treatment resulted in a significant downregulation of pro-inflammatory cytokine expression and an upregulation of T cell absolute counts in most cases, thus showing effectiveness of treatment in COVID-19.

## 6 Limitations of the study

This study has some limitation. Firstly, small number of participants due to not meeting the inclusion criteria, as well as prompt initiation of therapy not allowing for sampling prior to the treatment. Secondly, with the limited number of participants, it is difficult to evaluate risk factors for severity and mortality with multivariable-adjusted models. Finally, statistical results and p-values should be interpreted cautiously, as non-significant p-values do not necessarily rule out the difference between patients with moderate and severe disease.

## Data availability statement

The raw data supporting the conclusions of this article will be made available by the authors, without undue reservation.

## Ethics statement

The studies involving human participants were reviewed and approved by Ethical Committee at the Medical University of Bialystok - APK.002.6.2021. The patients/participants provided their written informed consent to participate in this study.

## Author contributions

DM, AP-K, and KG contributed to conception and design of the study. DM, AS, and KG have performed the experiments, acquired and analyzed the data. DM, AP-K, KG, MM, and RF prepared the manuscript. All authors contributed to the article and approved the submitted version.
