# Supplementary material for: Effect of antiviral and immunomodulatory treatment on a cytokine profile in patients with COVID-19
Source: Front Immunol. 2023 Jul 6;14:1222170. doi: 10.3389/fimmu.2023.1222170 (PMC10358833; doi:10.3389/fimmu.2023.1222170)
Supplement: Supplementary file 1 [file DataSheet_1.docx]

Supplementary Material

Effect of antiviral and immunomodulatory treatment on a cytokine profile in patients with COVID-19.

Diana Martonik, Anna Parfieniuk-Kowerda, Aleksandra Starosz, Kamil Grubczak, Marcin Moniuszko, Robert Flisiak

*** Correspondence:** Diana Martonik, diana.martonik@umb.edu.pl

# Supplementary Figures and Tables


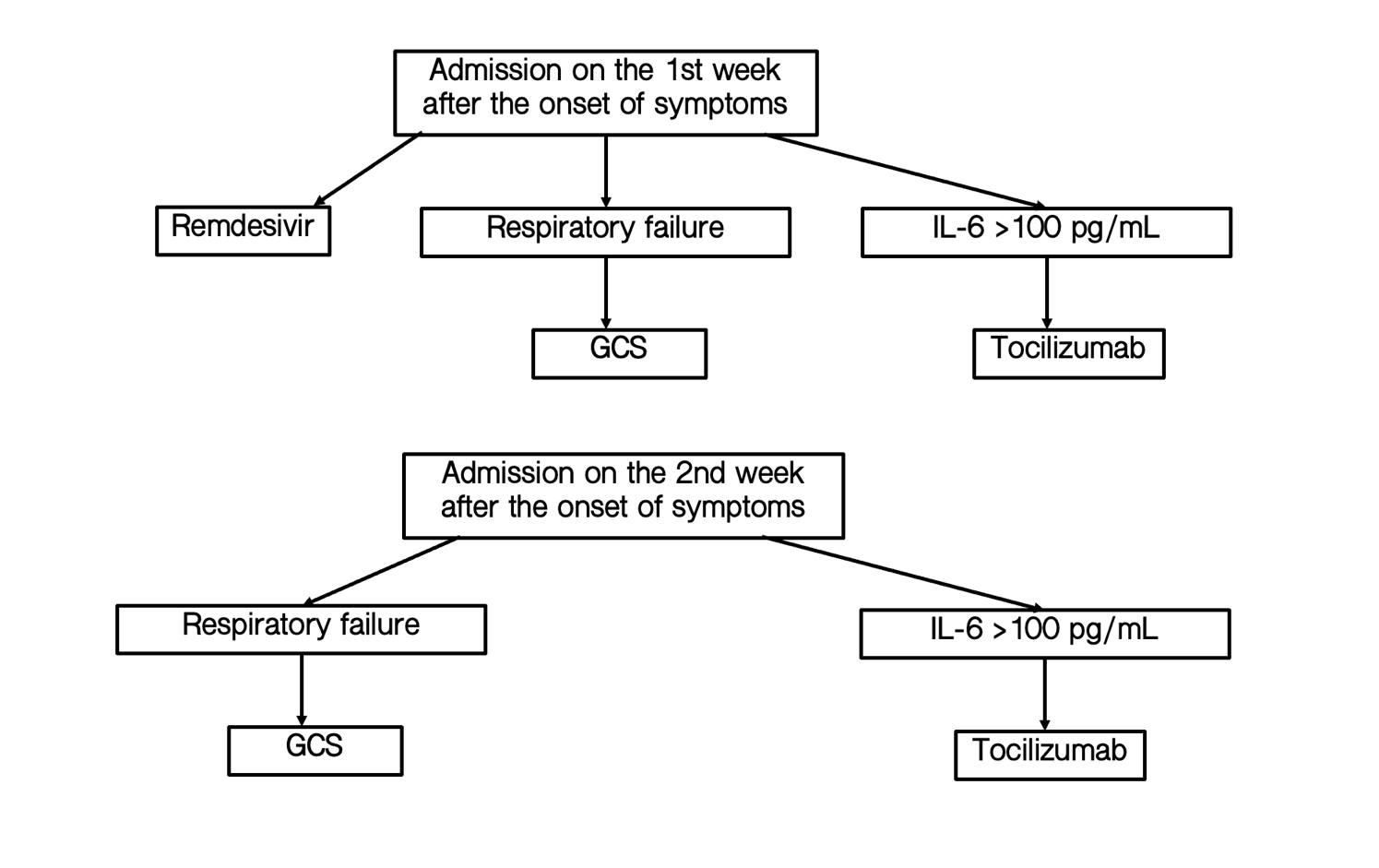


Supplementary Figure 1. Simplified therapeutic management with the reference to the time of patients admission from the onset of the symptoms.

| **Variables** | **Moderate cases (n=35)** | **Severe cases (n=9)** | **p** |
| --- | --- | --- | --- |
| Age, y | 60.0  (50.0-68.5) | 67.0  (57.0-73.0) | 0.354 |
| Gender, male | 23 (65.7%) | 9 (100.0%) | 0.002* |
| BMI, kg/m^2^ | 30.5  (27.6-34.9) | 29.0  (24.8-32.1) | 0.476 |
| SpO_2_, % | 92.0  (86.5-94.0) | 82.0  (78.0-88.0) | 0.004* |
| Time from onset, days | 7.0  (5.5-10.0) | 7.0  (5.0-9.0) | 0.481 |
| Comorbidities  Hypertension, n (%)  Diabetes, n (%)  Asthma, n (%)  Dyslipidaemia, n (%) | 20 (57.1%)  4 (11.4%)  2 (5.7%)  7 (20.0%) | 5 (55.6%)  3 (33.3%)  0 (0.0%)  1 (11.1%) | 0.932  0.109  0.463  0.537 |
| Symptoms  Fever, n (%)  Cough, n (%)  Dyspnoea, n (%)  Fatigue, n (%) | 27 (77.1%)  23 (65.7%)  19 (54.2%)  19 (54.2%) | 5 (55.5%)  5 (55.5%)  5 (55.5%)  5 (55.5%) | 0.195  0.572  0.946  0.946 |
| Lung involvement in CT scan on admission, % | 40.0  (30.0-60.0) | 50.0  (40.0-80.0) | 0.196 |
| Data represented as number or median (IQR). BMI = body mass index, SpO_2_ = oxygen saturation, * = statistical significance | | | |

Supplementary Table 1. General characteristics of moderate and severe cases.

| **Variables** | **Normal range** | **Moderate cases (n=35)** | **Severe cases (n=9)** | **p** |
| --- | --- | --- | --- | --- |
| ALT, IU/L | <31.0 | 38.0  (23.3-59.8) | 48.0  (37.0-86.0) | 0.289 |
| AST, IU/L | <32.0 | 51.0  (44.3-66.0) | 78.0  (44.3-135.5) | 0.157 |
| CRP, mg/dL | <5.0 | 84.7  (51.5-127.1) | 106.7  (88.0-175.9) | 0.111 |
| PCT, mg/dL | <0.05 | 0.08  (0.05-0.13) | 0.14  (0.09-0.54) | 0.097 |
| D-dimers, ng/mL | <500.0 | 853.0  (638.0-1377.0) | 1272.0  (814.0-1495.0) | 0.503 |
| Fibrinogen, mg/dL | 200.0-400.0 | 623.0  (527.0-758.0) | 695.5  (629.0-780.3) | 0.401 |
| LDH, U/I | 135.0-214.0 | 447.0  (338.3-580.5) | 635.0  (498.3-777.3) | 0.040* |
| Leukocyte count, x10^9^/L | 4.0-10.0 | 5.5  (4.7-8.4) | 6.0  (4.4-8.7) | 0.673 |
| Neutrophil count, x10^9^/L | 1.6-7.2 | 4.2  (3.1-7.4) | 4.4  (3.2-7.3) | 0.846 |
| Lymphocyte count, x10^9^/L | 0.8-4.7 | 0.9  (0.7-1.2) | 0.8  (0.5-0.8) | 0.054 |
| Lymphocytes, % | 18.0-48.0 | 13.4  (9.8-23.9) | 12.4  (8.8-15.5) | 0.429 |
| Haemoglobin, g/dL | 12.0-16.0 | 14.9  (13.4-15.4) | 14.7  (13.7-15.4) | 0.964 |
| Platelet count, x10^9^/L | 130.0-350.0 | 188.0  (156.0-268.5) | 145.0  (119.0-194.0) | 0.244 |
| INR | 0.8-1.2 | 1.2  (1.1-1.2) | 1.1  (1.1-1.2) | 0.570 |
| Data represented as number or median (IQR). ALT = alanine aminotransferase, AST = aspartate aminotransferase, CRP = C-reactive protein, INR = international normalized ratio, LDH = lactate dehydrogenase, PCT = procalcitonin, * = statistical significance  Supplementary Table 2. Laboratory findings in moderate and severe cases. | | | | |
